# Non-invasive detection of high gamma band activity during motor imagery

**DOI:** 10.3389/fnhum.2014.00817

**Published:** 2014-10-16

**Authors:** Melissa M. Smith, Kurt E. Weaver, Thomas J. Grabowski, Rajesh P. N. Rao, Felix Darvas

**Affiliations:** ^1^Graduate Program in Neuroscience, Department of Neurobiology and Behavior, University of WashingtonSeattle, WA, USA; ^2^Center for Sensorimotor Neural Engineering, University of WashingtonSeattle, WA, USA; ^3^Department of Computer Science and Engineering, University of WashingtonSeattle, WA, USA; ^4^Department of Radiology, University of WashingtonSeattle, WA, USA; ^5^Department of Neurology, University of WashingtonSeattle, WA, USA; ^6^Department of Neurological Surgery, University of WashingtonSeattle, WA, USA

**Keywords:** high gamma, EEG, fMRI, motor imagery

## Abstract

High gamma oscillations (70–150 Hz; HG) are rapidly evolving, spatially localized neurophysiological signals that are believed to be the best representative signature of engaged neural populations. The HG band has been best characterized from invasive electrophysiological approaches such as electrocorticography because of the increased signal-to-noise ratio that results when by-passing the scalp and skull. Despite the recent observation that HG activity can be detected non-invasively by electroencephalography (EEG), it is unclear to what extent EEG can accurately resolve the spatial distribution of HG signals during active task engagement. We have overcome some of the limitations inherent to acquiring HG signals across the scalp by utilizing individual head anatomy in combination with an inverse modeling method. We applied a linearly constrained minimum variance (LCMV) beamformer method on EEG data during a motor imagery paradigm to extract a time-frequency spectrogram at every voxel location on the cortex. To confirm spatially distributed patterns of HG responses, we contrasted overlapping maps of the EEG HG signal with blood oxygen level dependence (BOLD) functional magnetic resonance imaging (fMRI) data acquired from the same set of neurologically normal subjects during a separate session. We show that scalp-based HG band activity detected by EEG during motor imagery spatially co-localizes with BOLD fMRI data. Taken together, these results suggest that EEG can accurately resolve spatially specific estimates of local cortical high frequency signals, potentially opening an avenue for non-invasive measurement of HG potentials from diverse sets of neurologically impaired populations for diagnostic and therapeutic purposes.

## INTRODUCTION

The HG band (70–150 Hz) is a rapidly evolving, spatially localized signal (Crone et al., 1998; Miller et al., 2009) that is thought to be associated with local neuronal processing (Manning et al., 2009; Miller et al., 2009). This high frequency activity has been found across the cortex reflecting local computation across a number of functional domains including sensory processing, attention, memory, and movement control. Additionally, invasive studies with electrocorticography (ECoG) have shown that motor imagery elicits HG activity in the motor cortex (Miller et al., 2010). Combined with the rapidly evolving, spatially constrained nature of the signal this makes HG band responses an attractive candidate for BCI control. Unfortunately, HG activity overlaps entirely with the spectral bandwidth of muscle activity (20–300 Hz) leading to artifacts from muscle activity in non-invasive EEG recordings from the scalp. In addition, the high frequency band has a low signal-to- noise ratio (SNR) in EEG recordings due to the attenuation and smearing of electrical potentials when they diffuse through the intervening tissues (dura, skull, and scalp) to the surface recording electrode (Buzsáki et al., 2012). Therefore, the bulk of the work done to date on HG activity has used invasive recording modalities where the SNR is greater and muscle artifacts almost non-existent.

It has recently been shown that HG activity can be detected non-invasively by EEG and magnetoencephalography (MEG). HG activity was found in the primary motor cortex prior to finger movements in EEG recordings (Ball et al., 2008; Darvas et al., 2010) and MEG studies have demonstrated that high frequencies peak around 70–80 Hz in the primary motor cortex, with a bandwidth of ∼40 Hz, during self-paced movements of the upper and lower limbs (Cheyne et al., 2008; Dalal et al., 2008). These peak frequencies and bandwidth were found to vary across individuals and the limb that is moved.

During motor execution, as well as mental motor imagery, event-related desynchronization (ERD) can be detected both invasively and non-invasively in the mu (8–13 Hz) and the beta (18–30 Hz) frequency ranges (McFarland et al., 2000; Neuper and Pfurtscheller, 2001; Neuper et al., 2005). High frequency signals in motor cortex during motor execution and imagery have been detected using invasive modalities including ECoG (Miller et al., 2010), but have not yet been reported for non-invasive recording methods during motor imagery. However, other studies have shown, e.g., visual cortex activity in the HG range during a mental rotation task(de Lange et al., 2008), using MEG.

In order to examine the relationship between fMRI and the underlying neurophysiological responses several studies have compared local field potentials (LFPs), single-unit and multi-unit spiking activity in functionally relevant areas of cortex with BOLD signal responses (Logothetis et al., 2001; Mukamel et al., 2005; Niessing et al., 2005). These studies found a positive correlation between the high frequency power changes in the LFPs and BOLD signal changes. However, the cortical regions surrounding the functionally relevant areas were not examined and it is unclear whether these regions have neurophysiological correlates as well. It may be expected that they do because typical BOLD activity maps display large BOLD signal changes in cortical areas known to be related to the behavior being performed, as well as weaker, more variable signal changes in the surrounding areas (Rombouts et al., 1997; Saad et al., 2003).

Two recent studies (Conner et al., 2011; Hermes et al., 2012) examined the correlation of the BOLD signal and spectral power changes measured by ECoG. Hermes et al. (2012) found that spectral power increases in the high frequency range co-localized with spatially focal BOLD peaks on primary sensorimotor areas, while Conner et al. (2011) showed positive correlation of bold with HG in pre and post-central areas (i.e., covering motor area) and negative correlations with the beta band.

However, ECoG recordings only provide a limited neurophysiological correlation because they do not record from the whole brain. Non-invasive studies with simultaneous EEG and fMRI have reported negative correlations with BOLD in the low frequency (4–30 Hz) range of the spectrum (Yuan et al., 2010), but the spatial association between whole-brain HG band neural activity detected by EEG and the hemodynamic changes of the fMRI BOLD signal has not been reported.

Here we present an analysis of HG activity during motor imagery using non-invasive EEG in healthy subjects. The EEG inverse mapping results were contrasted with fMRI BOLD response for the same paradigm from the same subjects.

## MATERIALS AND METHODS

### SUBJECTS

Data were recorded from 10 healthy adult subjects (four males, mean age = 24.9 years, range = 20–30 years). Nine subjects were right-handed and one subject was left-handed. Subjects gave their informed consent according to the protocol approved by the Institutional Review Board (IRB) of the University of Washington.

### TASK

For both fMRI and EEG sessions, subjects were cued to imagine moving their fingers in a tapping sequence: pinch thumb to each digit once from proximal to distal and then ending the sequence by pinching thumb to ring-finger. Subjects imagined moving both their left and right hands. During the EEG session, subjects were seated in a recliner and four blocks of 25 right and 25 left hand trials were recorded, totaling 100 trials per hand. The subjects had their eyes open and fixated on a fixation cross or cue. Each trial consists of a rest period of 2 s, during which a fixation cross was shown. At the end of that rest period the fixation cross was changed to a written instruction, i.e., the cue, which was either “right” for right hand imagery or “left” for left hand imagery. The cue was shown for 3 s and then changed back to a fixation cross, which concluded the trial. During each block “left” and “right” cues were presented in random order, for a total of 50 cues per block.

### MRI/fMRI DATA ACQUISITION

Scanning was conducted at 3T (Philips Achieva) using an 8-channel head-coil. For source estimation and cortical surface reconstruction, T1-weighted 3-dimensional high-resolution multi-echo MPRAGE (MEMPRAGE) structural images (with a four echo read out with echo times starting at 2 ms and stepped every 2 ms) and two Fast low-angle shot (FLASH) sequences (Haase et al., 1986) starting at both TE 5 and 30 ms respectively, (each with an echo train of 6, stepped every 2 ms) were acquired. All sequences were reconstructed into a 1 × mm × 1 × mm 1 × mm tissue space before head model reconstruction.

Whole brain functional images for each task-based scan were collected using a gradient echo T2^∗^ weighted sequence (TE/TR = 21/2000 ms, matrix size 80×80). Subjects participated in a standard block-designed fMRI task of imagined movement, with randomly presented blocks of left and right imagery as well as rest. A total of 15 blocks were presented, with each conditional block being presented five times. No condition was repeated. Each block consisted of 10 visually presented cues (left, right, or rest) in the center of the screen for 2 s followed by a 2 s inter-trial interval. Each block lasted for 40 s. The experiment began and terminated with a 40 s rest period.

### EEG/EMG DATA ACQUISITION

**FIGURE 1:**
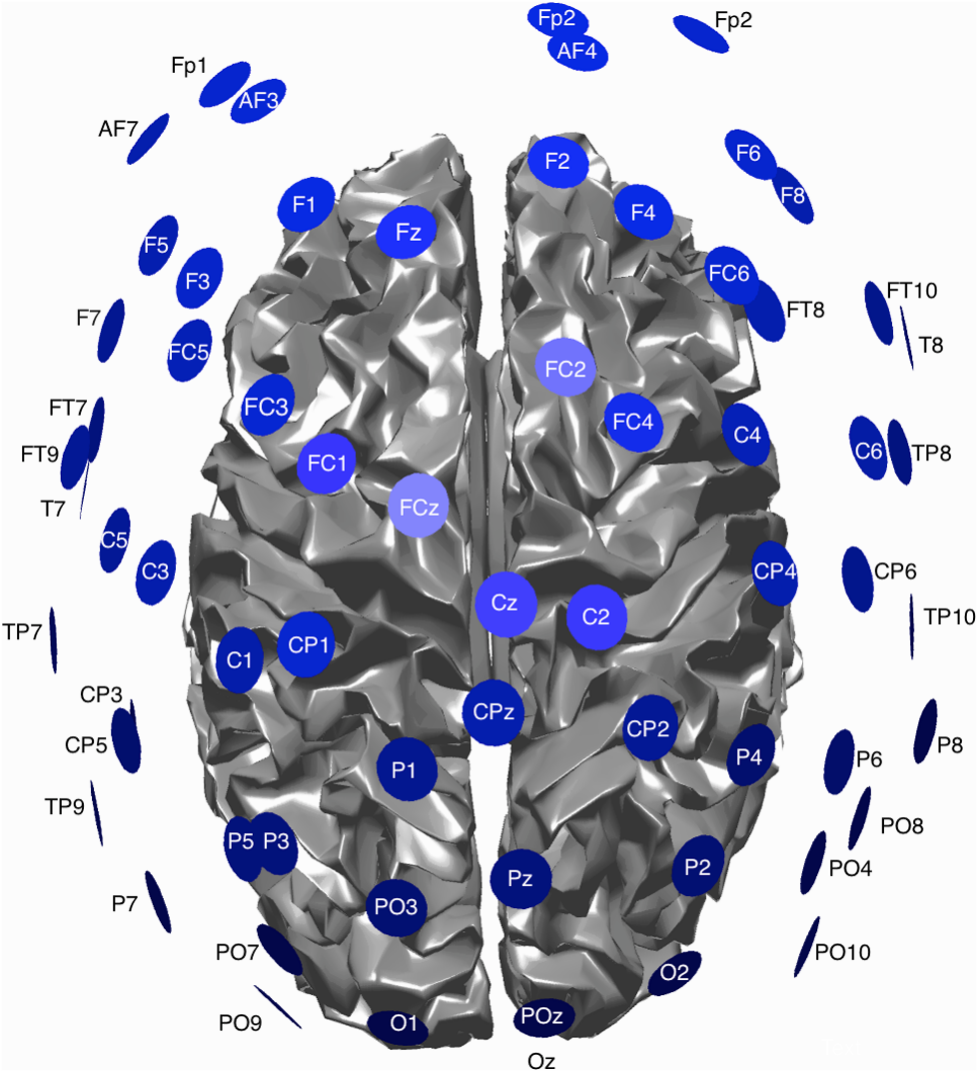
**Schematic view of the EEG montage and cortical surface used for EEG data acquisition**.

EEG data was continuously recorded from 54 electrodes [BrainProducts 64-channel actiCAP (BrainProducts, Gilching, Germany)] during each block. The actiCap has a subset of electrodes based on the 10–20 system. A schematic of the montage is shown in **Figure 1**. Data was sampled at 1200 Hz, using four GugerTec (GugerTec, Graz, Austria) EEG amplifiers recorded in DC, from -250 to +250 mV. Impedance values were kept below 20 kΩ. In parallel, we recorded at the same sampling rate the EMG from the flexor indices from both hands and EOG (electro-oculogram) in bipolar configurations.

A 3D localizer (Patriot, Polhemus, Colchester, VT, USA) was used to determine the electrode positions for each subject as well as the positions of three anatomical landmarks: nasion, and the left and right pre-auricular points.

### EEG/EMG DATA SEGMENTATION

For each block, data was segmented into 5 s long segments, with time 0 s centered at the presentation of the cue, i.e., “left” or “right” hand imagery, resulting in a within-trial time axis ranging from -2 to 3 s. The motor imagery was taking place anywhere between 0 and 3 s. The same segmentation was applied to EMG and EOG data.

### HEAD MODELING

A 3D structural headmodel was created for each participant by averaging across all acquired echo times within the MEMPRAGE scan and incorporating two FLASH sequences (flip angle = 5 and 30°). We used the FreeSurfer ^1^ reconstruction software for an automated segmentation of the MR into separate tissue types and boundary surfaces, specifically *scalp, outer skull, inner skull, and white matter/gray matter interface.* The latter served as a source space for EEG source reconstruction. The BrainStorm software package (Tadel et al., 2011 ^2^) was then used to compute a realistic BEM for each subject. The final BEM output was used to create a forward model for each subject, which is a requirement for any inverse mapping of activity from the EEG sensors to the cortex. Scalp electrode positions, measured with a Polhemus FastTrak device (Polhemus, Colchester, VT, USA), were co-registered with the MR images and thus with the headmodel through three anatomical landmarks: nasion, left pre-auricular point, and right pre-auricular point.

### SOURCE MAPPING

We used a linearly constrained minimum variance (LCMV) beamformer to map from the EEG channel domain onto the white/gray matter interface. Similar to a minimum norm least squares (MNLS) estimate mapping, the LCMV provides a transfer matrix from channel domain to source domain, which is essential for fast computation of source maps, but produces more focal results (Darvas et al., 2004). ECoG studies (Crone et al., 1998; Miller et al., 2007) have shown HG to be typically focal in nature and thus the LCMV provides a more accurate mapping of this focal source type than, e.g., the MNLS method. Similar to (Darvas et al., 2013), we compute a map of cortical activity for a specific frequency from a wavelet transform of the data, using the complex Morlet wavelet for the time-frequency decomposition. The beamformer weights were computed based on the broadband signal, which yields a single set of channel weights for each cortical source location, which are then applied to each of the complex wavelet transformed data. Due to the linearity of the mapping transform, the complex wavelet coefficients for a specific frequency for the channel data can be mapped into source space by multiplication of the transfer matrix to the time by channel vector of complex coefficients, i.e.,:

$$j_{i}\,\left(t,f\right)=T\,\;d_{i}\,\left(t,f\right),$$

where *d* is the complex wavelet transform of recorded data at time *t* and frequency *f*, *T* is the transfer matrix and *j* is the resulting cortical map of complex coefficients for the *i*th trial. The average time varying power or amplitude is then computed as:

$$p\,\left(t,f\right)=\sum_{i=1}^{N}{\left|j_{i}\,\left(t,f\right)\right|}^{2}\,\;\left(p\,o\,w\,e\,r\right)\,\;o\,r$$

$$a(t,f)=\overset{N}{\underset{i=1}{\sum }}\left|j_{i}\left(t,f\right)\right|\,\;\;\;(amplitude),$$

where N is the number of trials. Here we compute amplitudes, to reduce the sensitivity to outliers. We normalize the resulting cortical amplitude map with respect to a baseline interval (-1 to 0 s) to equalize the amplitude differences across voxels of the map, by computing *Z*-scores (see Bar et al., 2006) for similar application). The *Z*-scores of the amplitude time series represent changes in a given frequency relative to the baseline, measured in units of the standard deviation (over time) of the baseline. Unlike a relative percent change measure, which is frequently used in ERD/S analysis, the *Z*-scores take into account the variability of the baseline and thus provide a more realistic assessment of the relative change.

### STATISTICAL ANALYSIS

#### fMRI/MRI

fMRI data processing was carried out using FEAT (FMRI Expert Analysis Tool) Version 5.98, part of FSL (FMRIB’s Software Library ^3^). The preprocessing pipeline included motion correction, high-pass temporal filtering for removal of liner drift, a spatial filter of 5 mm full-width half maximum (FWHM) and a pre-whitening filter to remove signal autocorrelations throughout the time-course. BOLD responses were estimated on an individual subject basis by applying a box-car, general-linear model design with a standard hemodynamic response convolution. Whole-brain BOLD activity was contrasted between active (left or right) and rest periods, converted into *Z*-scores and with a threshold at *Z* > 2.3 (uncorrected). Clusters of significant activity were masked into regions of interest (ROIs). For each participant, all functional data sets were co-registered into native MPRAGE space using a rigid-body (6 degrees of freedom) registration and trilinear interpolation.

#### EEG

EEG signals, especially in the HG range, are highly susceptible to noise originating from non-cortical sources. Of particular concern is that of muscle activity (EMG), which overlaps the HG frequency range and exceeds cortically generated HG power by orders of magnitude. EMG artifacts can arise from neck muscles, facial muscles, particularly the jaws and are difficult to control. During the course of the experiment, inevitably these artifacts will contaminate a number of trials, even in the overall absence of gross motion or position shifts of the subject. Since the sources of these artifacts are random, removal of these artifacts from the signal of interest based on stable spatial patterns across the sensors is difficult. In order to control for these artifacts, we remove any contaminated trials from the study. Unlike cortical sources, the EMG spectrum increases in power over a much wider range, thus excessive increase in power above 100 Hz, very large increases in HG power (>99 percentile of the overall HG power over time) or synchronous increases in multiple channels were identified as artifacts. Artifact contamination in the analysis of event related synchronization (ERS) is additionally confounded by the fact that we average over powers, i.e., the errors in the average due to artifacts do not cancel out. Therefore significant changes in mean amplitude or power, e.g., in the time evolution of HG power can easily be attributed to a single artifact contaminated trial.

To ensure the stability (across trials) and significance of HG changes during motor imagery, we applied a permutation test. We tested for a significant increase of HG amplitude during the period of the paradigm where the subject was instructed to imagine movement. Here we consider an interval from *t* = 0 (presentation of instruction, i.e., cue, to imagine either left or right hand movement) to 1 s. The permutation test has the advantage of not having to make any assumptions about the null hypothesis, i.e., HG activity during periods of non-motor imagery. Furthermore, it allows us to test for non-linear statistics of the data (here the maximum HG power change over the cortical volume and over time) and it is less sensitive to outliers in single trials, thus ensuring trial-to-trial stability of the average HG power. For the permutation test, which is described in detail in (Pantazis et al., 2005), we randomly exchange a one second interval from (-1 to 0 s) of the base line with the signal interval (0 to 1 s) for half of all the trials, before computing the average time varying amplitude for a given frequency across trials.

Such a surrogate signal is computed for every voxel in our cortical map. By repeating this step *n* times, we can build up a histogram for any statistic we wish to compute on the amplitude *Z*-scores. Since we postulate an increase in HG amplitude in the signal interval, we chose the maximum of the *Z*-score over the signal period. Since motor imagery is subject initiated, we expect onset times of any HG changes to vary substantially between subjects. By taking the maximum over the signal interval, our statistic is independent of individual variation in that period. Also, for the permutation test, we avoid having to correct resulting *p*-values of the test for multiple comparisons across, e.g., different time points. Non-invasive, scalp-based approaches (Cheyne et al., 2008; Darvas et al., 2010, 2013) have shown that HG activity in the motor system is relatively narrow band and can vary across individuals. Therefore, for the permutation test, we test over the whole band from 70 to 100 Hz in 1 Hz steps and again, choose the maximum *Z*-score across frequencies. Finally, due to the variant spatial resolution of the inverse mapping, we also select the maximum across all voxels of the map, i.e., the whole cortex. By building a histogram over the maximum in time, frequency and space, we avoid having to correct the resulting *p*-values for any multiple comparisons. The permutation test also implicitly examines trial-to-trial stability of the average. That is, since any outlier trial with large HG power in the signal interval, which could cause the average HG power to be high as well, has a 50% chance of being permuted with a baseline segment and would skew the baseline HG power distribution in the same manner, the resulting average power would consequently no longer be in the tail end of the baseline distribution. Therefore, increases in the average HG power over trials in the signal period due to outliers will be tested as non-significant

#### EMG/EOG data processing

EMG and EOG are sources of noise in the HG band and can exceed the cortical signal considerably. EOG electrodes were placed approximately 1 cm above the left and right brows and 1 cm below left and right eyes. Through the permutation test on the EEG data, we can make sure that any significant HG effects observed in the data are not due to, e.g., transient, non-stimulus induced HG activity originating from either EMG or EOG. However, any stimulus-induced, systematic EMG or EOG activity that would introduce an artifact into the EEG would still pass the permutation test. In order to rule out such systematic contamination, we compute the average EMG and EOG HG power over all trials. This was then subsequently entered into into the EEG source analysis to identify systematic increases in HG in the EMG and EOG channels. Since EMG and EOG are typically of broadband nature, in the range from 70 to 100 Hz, we band pass filtered both signal types for each subject and side and computed the mean amplitude from the Hilbert transform of the 70–100 Hz filtered signals. Because the human brain’s power spectrum recorded non-invasively hits the noise floor at around 100 Hz, this 70–100 Hz band pass filter was applied to EEG recordings.

#### Determining goodness of match between EEG HG and fMRI and its significance

In order to determine how well the HG EEG map and the fMRI match, we group the significant fMRI activity and significant HG activity (i.e., at *p* < 0.1) into contiguous clusters on the cortical surface. For each cluster we compute a centroid position and a mean spherical radius, i.e., the mean distance of each cluster member from the center. For each EEG and fMRI cluster, we compute the distance between modalities as the distance between the centroids minus the sphere-radius for each cluster. Negative distances, due to overlap of clusters, are set to zero. For all fMRI clusters, we select the distance to the nearest EEG cluster. If there are more fMRI clusters than EEG clusters, we select smallest distances, matched to the number of EEG clusters. A compound measure is formed by taking the median across these minimal distances. This will serve as our assessment of the goodness of match between solutions.

Using 3D distances instead of actual geodesic distances on the folded cortical surface is a simplification of the ground truth match between modalities. However, deviations from the “true” distance are expected to be small, as we expect this value to be dominated by gross mismatches on the order of several centimeters and our measure merely serves as proxy for the goodness of match.

To compute statistical significance for our goodness-of-fit measures, we resampled the fMRI clusters on the cortical surface for each subject and condition (left or right imagery), where we keep the number of clusters and the cluster size constant between our permutation and the original. Thus we can build a histogram of goodness of match values for arbitrary fMRI solutions of similar shape to the original. A *p*-value, and thus a measure of the specificity of the actual match, is derived by comparing the actual measure vs. the resampled value. We generate 1000 resamples per subject and condition to provide for a sufficient accuracy of the computed *p*-values.

## RESULTS

### HG EEG ACTIVITY

**FIGURE 2:**
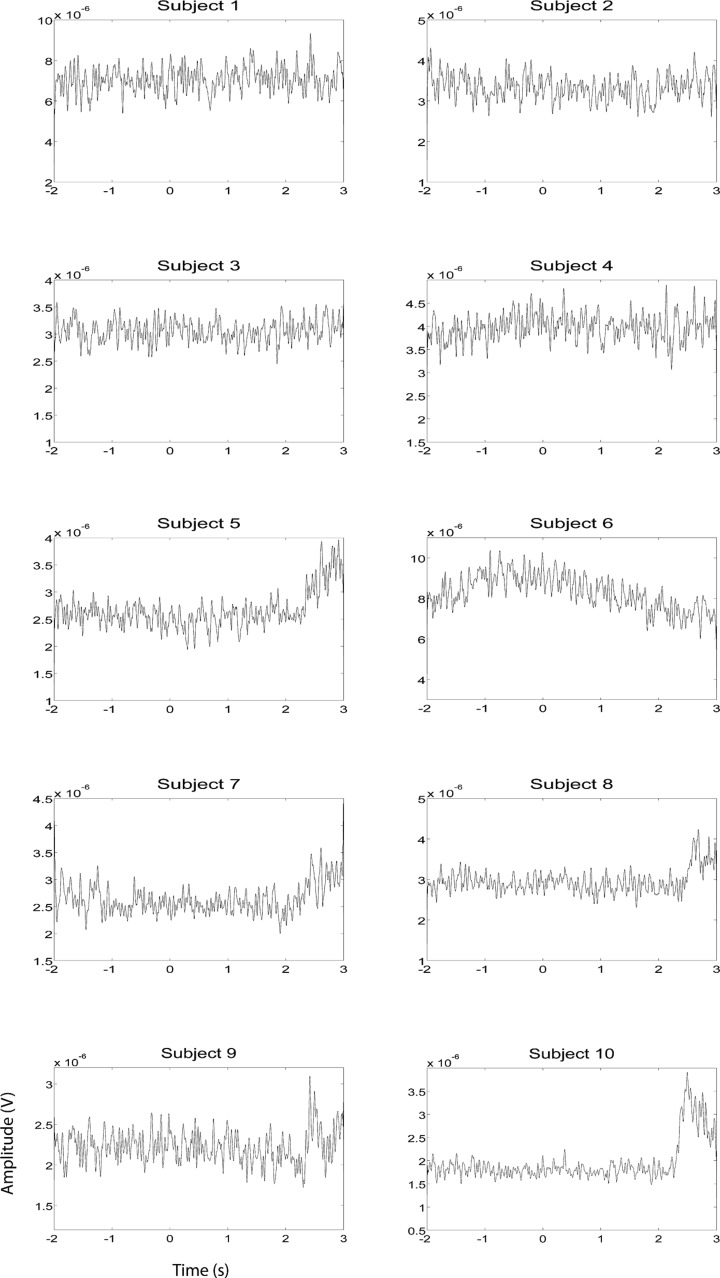
**EMG HG magnitude of the left hand during left hand motor imagery EEG recording sessions (*n* = 10).** EMG activity was averaged over all trials that were not contaminated by artifacts. Units of the EMG are in V.

**FIGURE 3:**
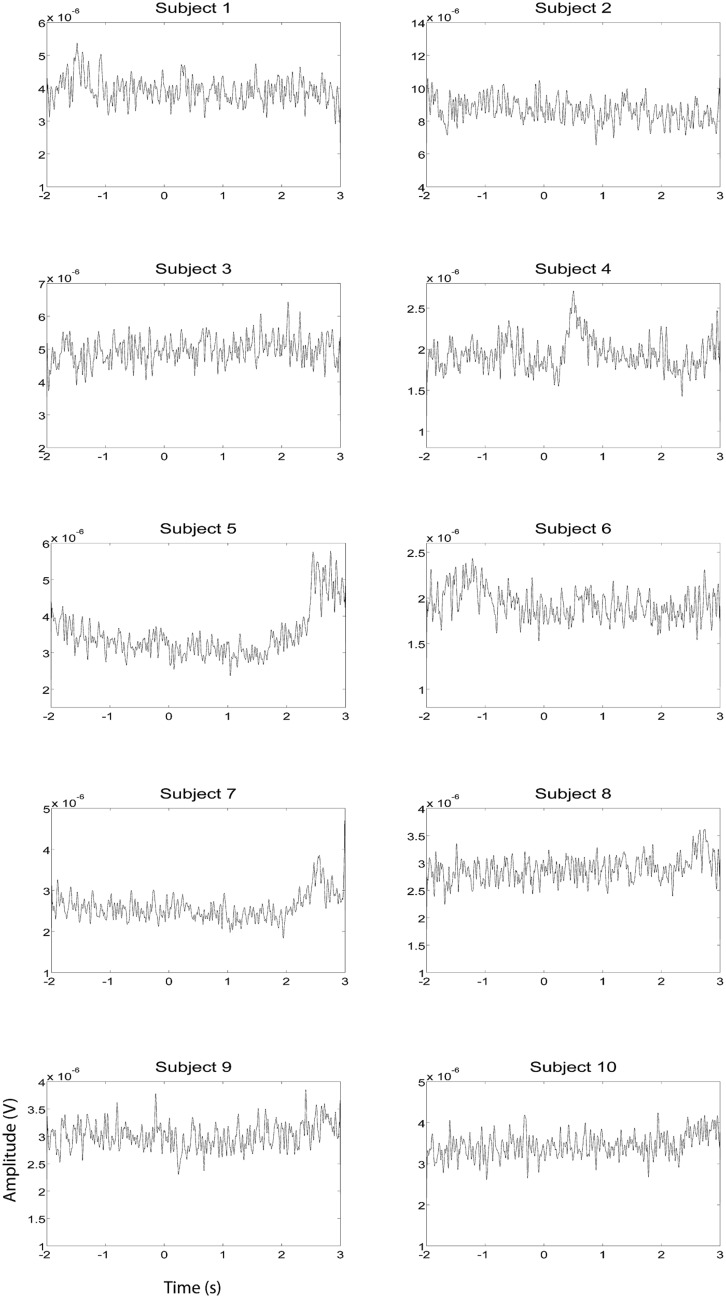
**EMG HG magnitude of the right hand during right hand motor imagery EEG recording sessions (*n* = 10).** EMG activity was averaged between all trials that were not contaminated by artifacts. Units of the EMG are in V.

**FIGURE 4:**
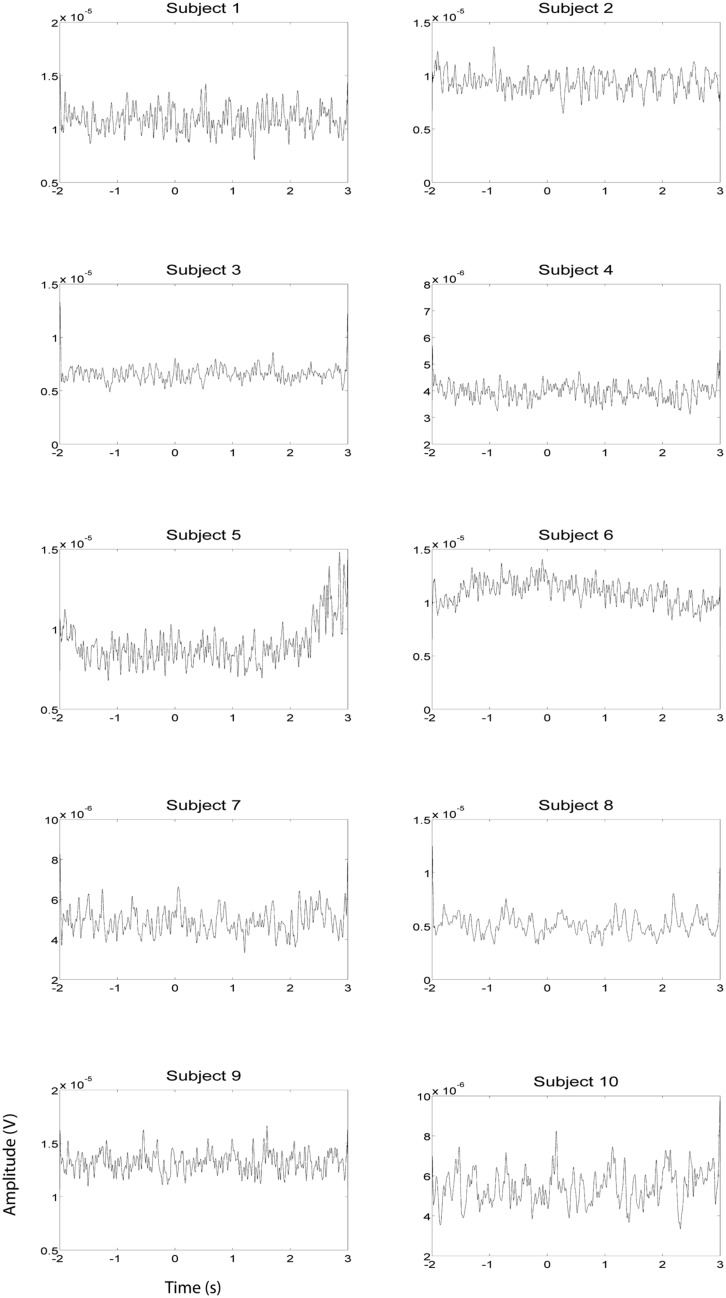
**EOG HG magnitude during left hand EEG motor imagery recording sessions (*n* = 10).** EOG activity was averaged over all trials that were not contaminated by artifacts. Units of the EOG are in V.

**FIGURE 5:**
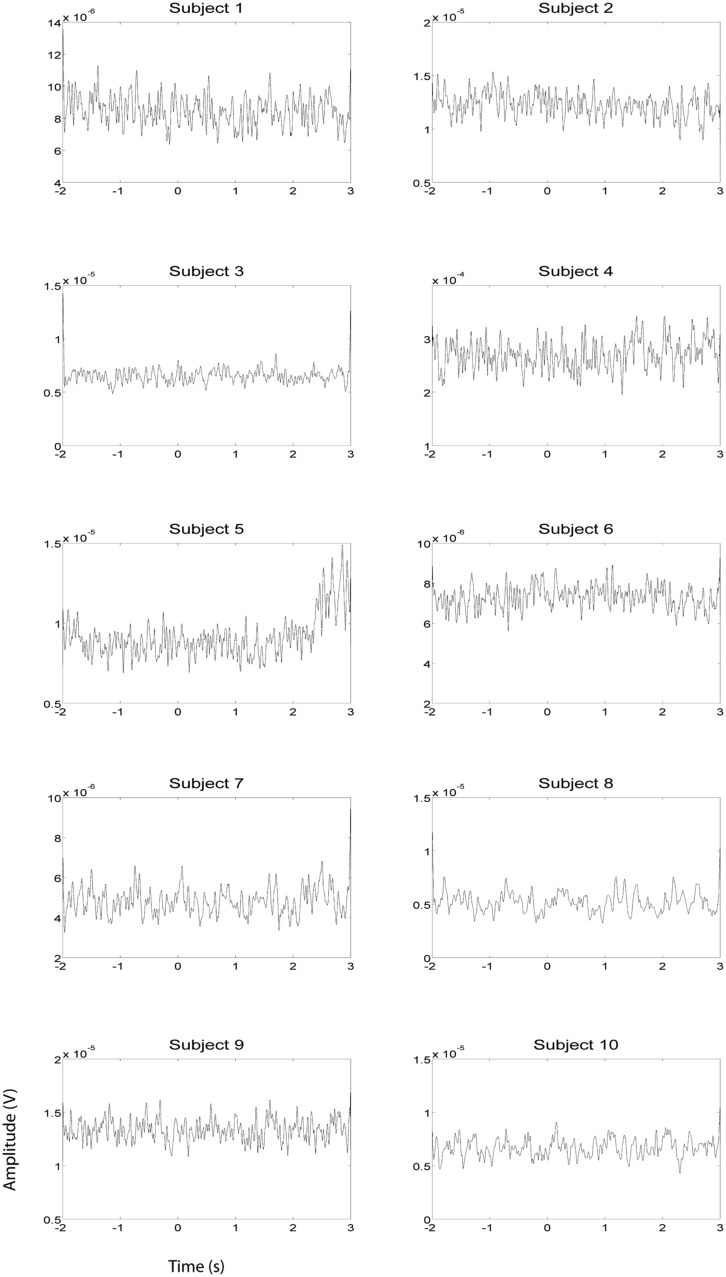
**EOG HG magnitude during right hand EEG motor imagery recording sessions (*n* = 10).** EOG activity was averaged over all trials that were not contaminated by artifacts. Units of the EMG are in V.

EEG and fMRI data were collected on separate days in the same group of ten subjects using identical behavioral protocols (see **Figure 1** for the EEG montage used). The only significant task induced EMG activity between 0 and 2000 ms after motor imagery cue onset was found in subject 4 (**Figures 2 and 3**). There was significant increase in EOG activity in subject five after 2000 ms, but also not overlapping with any cortical HG activity (**Figures 4 and 5**).

**FIGURE 6:**
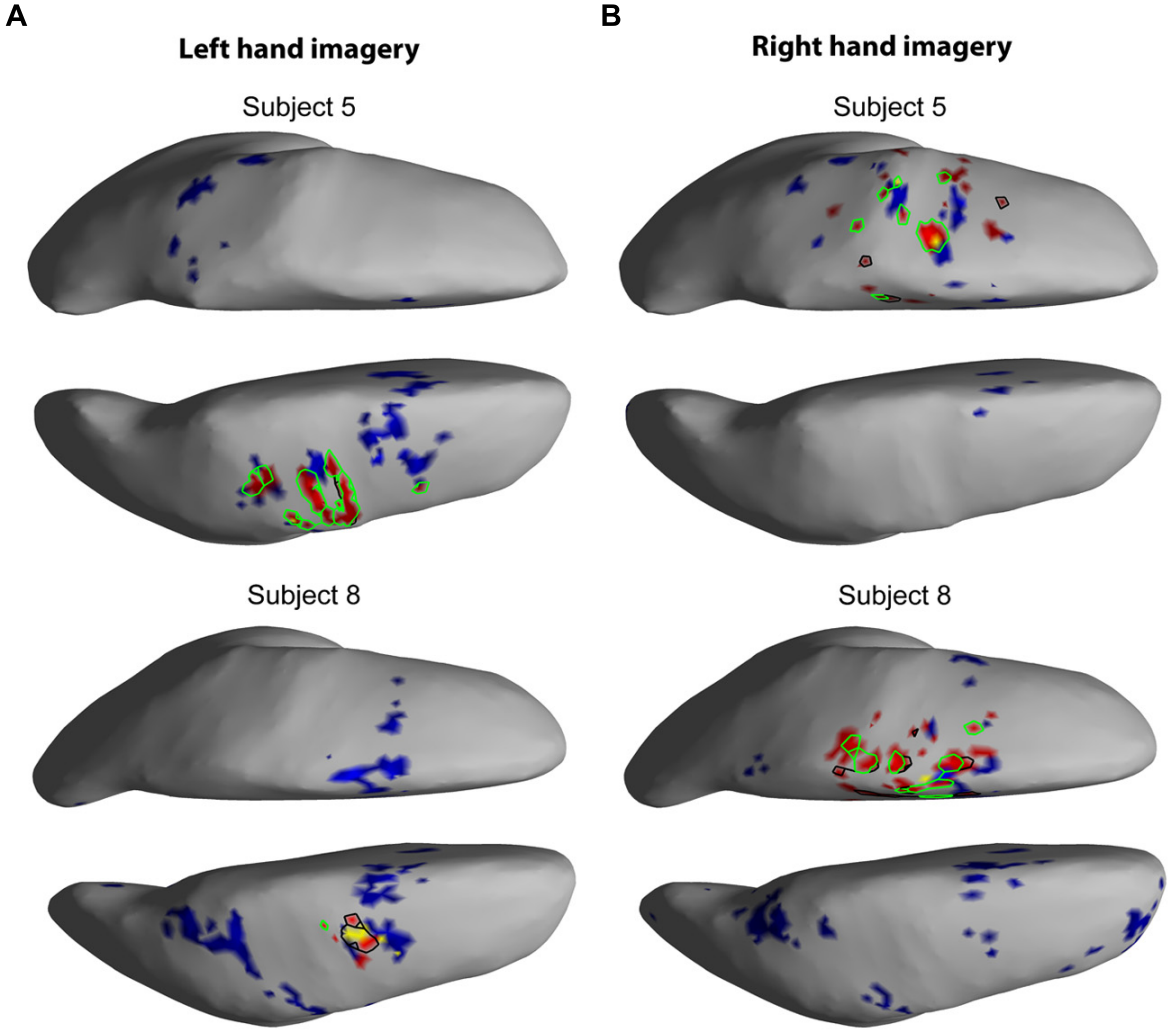
**EEG and fMRI BOLD activity mapped to realistic cortical headmodels of subjects 5 and 8.** The cortical surfaces have been smoothed for better visibility. Blue indicates significant fMRI BOLD activity at *Z*-score > 2.3. Red indicates significant EEG HG activity at Z > 7, the black contour line shows HG at *p* < = 0.1, the green contour line shows HG at *p* < = 0.05, and yellow colored areas show overlap between fMRI and EEG. **(A)** Cortical activity during left hand motor imagery. **(B)** Cortical activity during right hand motor imagery.

**FIGURE 7:**
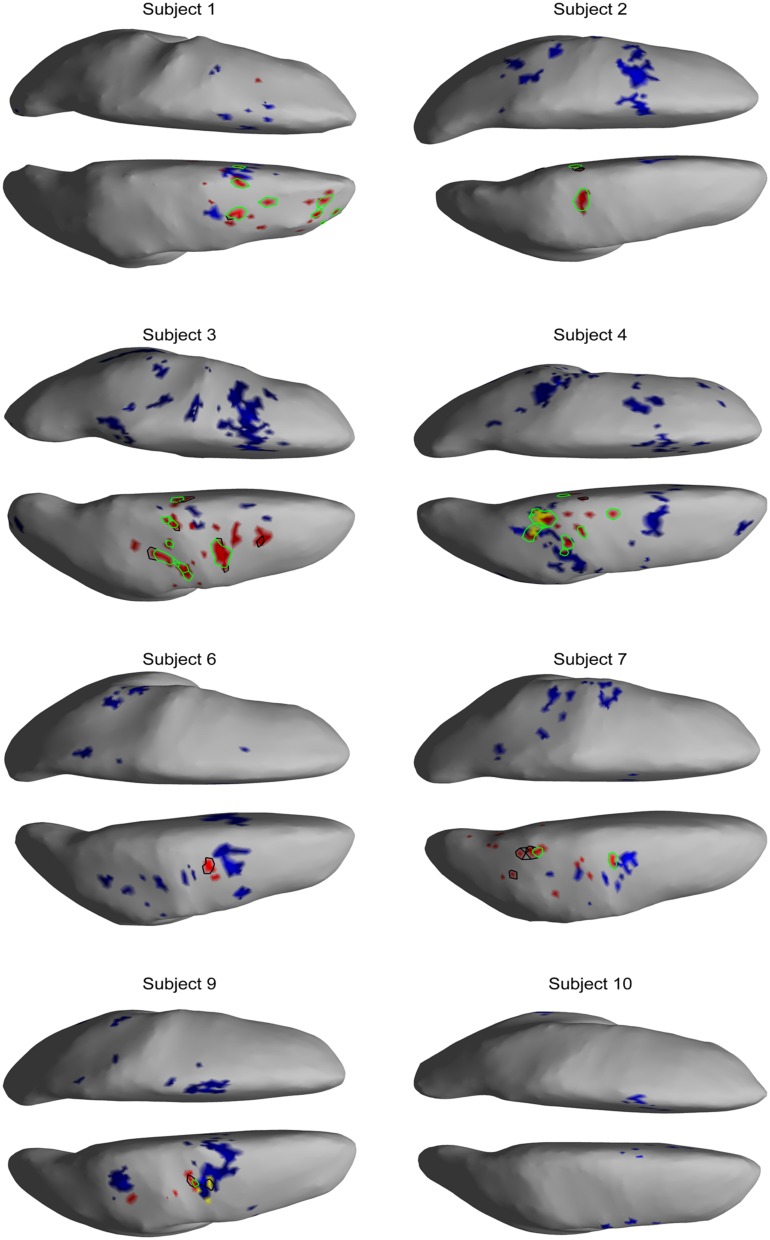
**EEG and fMRI BOLD activity mapped to realistic cortical headmodels of 7 of the remaining subjects during left hand motor imagery**.

**FIGURE 8:**
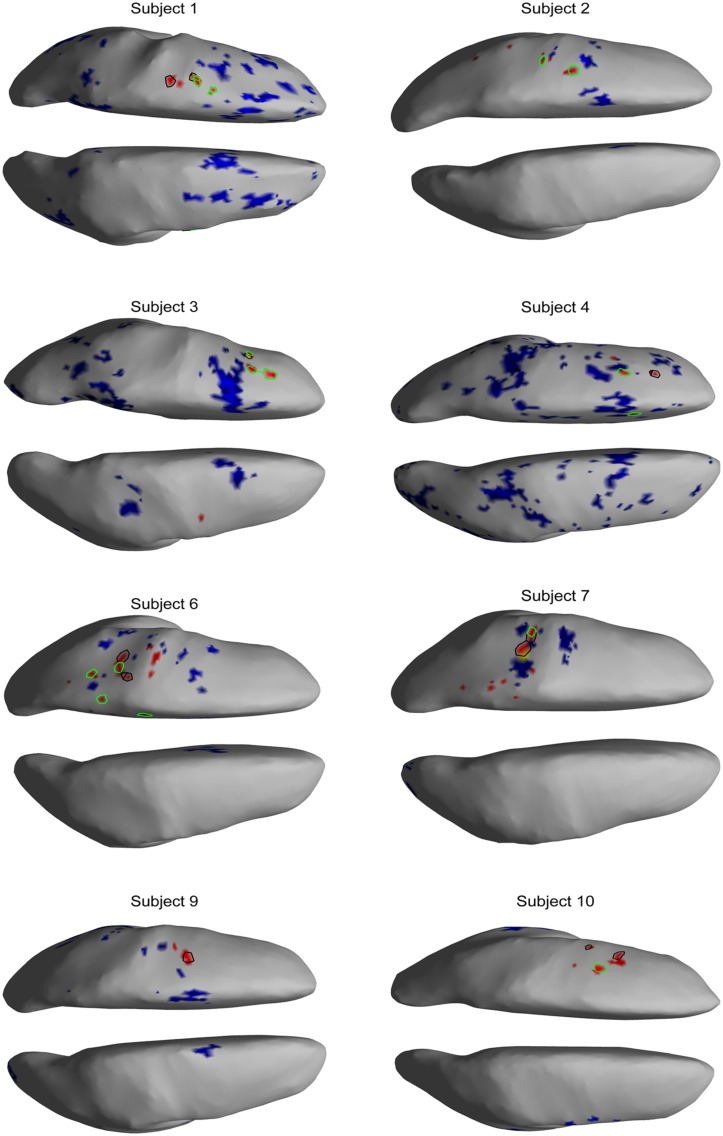
**EEG and fMRI BOLD activity mapped to realistic cortical headmodels of the eight remaining subjects during right hand motor imagery**.

The EEG results show significant power increases in the HG band during motor imagery (**Figure 6** for subjects 5 and 8. For the remaining eight subjects, see **Figures 7 and 8**). These power increases were spatially focal and mostly restricted to the sensorimotor areas of the contralateral cortex, but do cover other cortical areas, not typically associated with motor imagery as well. Single-subject fMRI activity, which is also shown in **Figures 5–8**, also does overlap in most subjects with the EEG reconstruction, but also shows unrelated activity. We find at least one cluster with *p* < 0.05 in 9 out 10 subjects for right hand imagery and in 8 out of 10 subjects for left hand imagery. Two subjects had only HG activity with *p* < 0.1 for one side (subject 6 for left hand imagery and subject #9 for right hand imagery). Subject #10 had no significant HG activity for left hand imagery.

**FIGURE 9:**
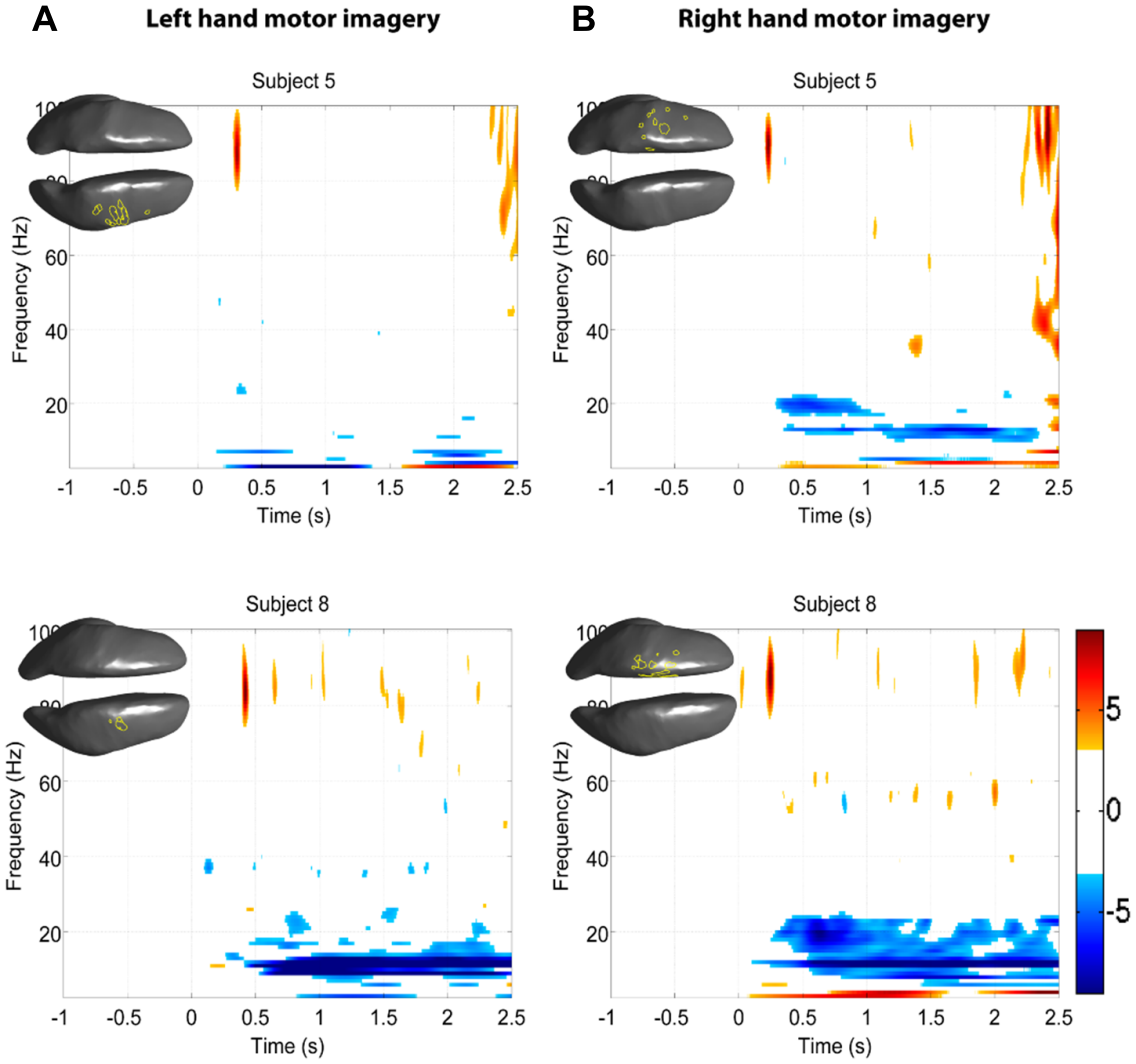
***Z*-score time-frequency maps for subjects 5 and 8 for the cortical regions indicated on the cortical surface displayed in the upper left corner.** The maps show that subjects have significant HG increases post-cue in a narrow band. **(A)** Time-frequency maps during left hand motor imagery. **(B)** Time-frequency maps during right hand motor imagery.

**FIGURE 10:**
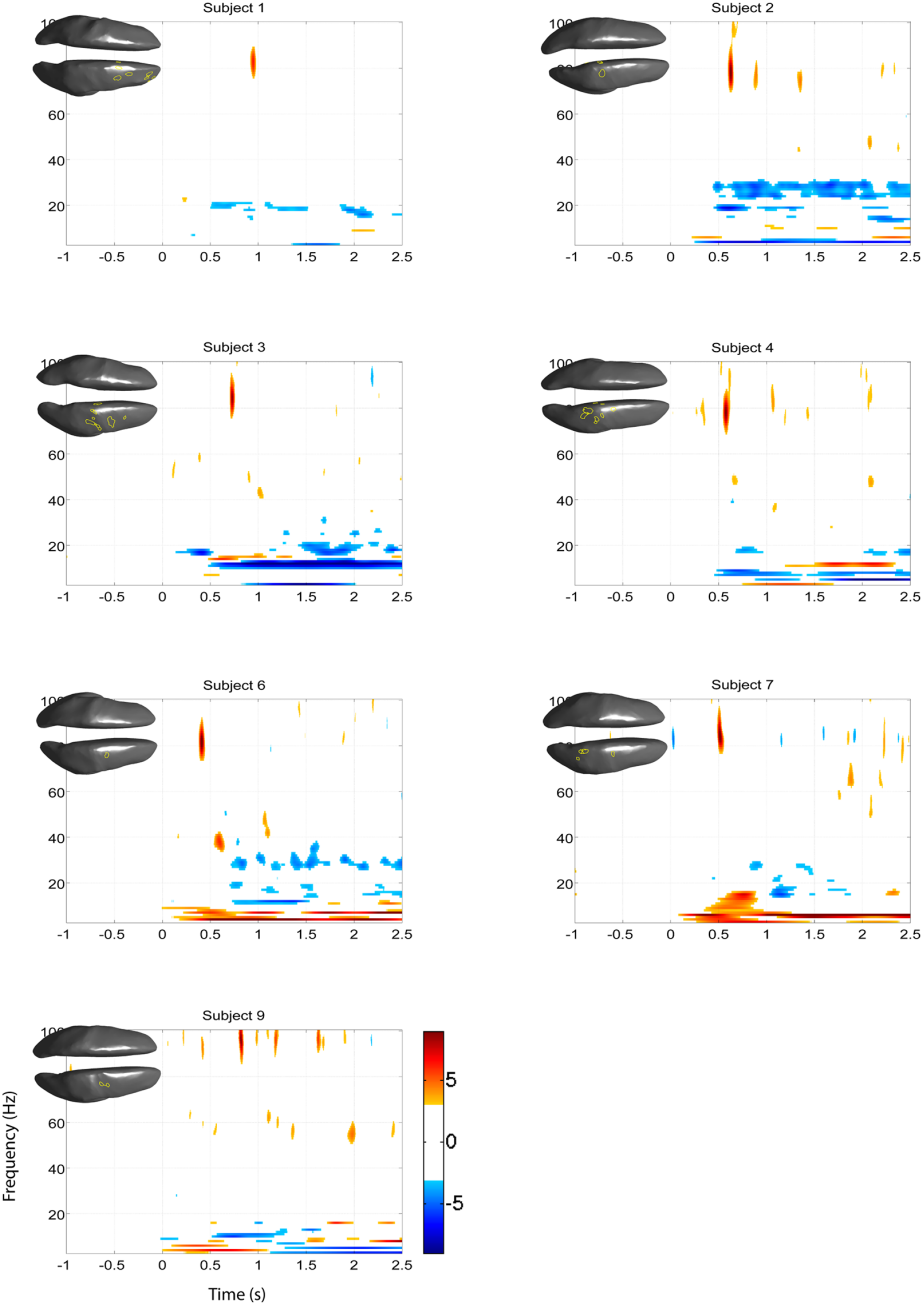
***Z*-score time-frequency maps during left hand motor imagery of the eight remaining subjects for the cortical regions indicated on the cortical surface displayed in the upper left corner.** The maps show that subjects have significant HG increases post-cue in a narrow band.

**FIGURE 11:**
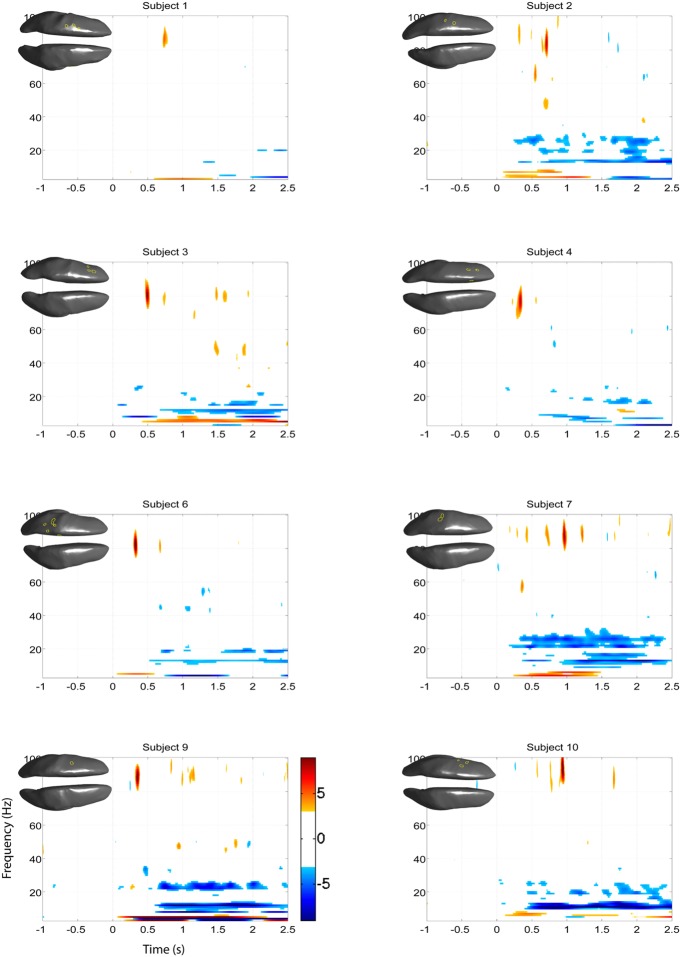
***Z*-score time-frequency maps during right hand motor imagery of the eight remaining subjects for the cortical regions indicated on the cortical surface displayed in the upper left corner.** The maps show that subjects have significant HG increases post-cue in a narrow band.

**FIGURE 12:**
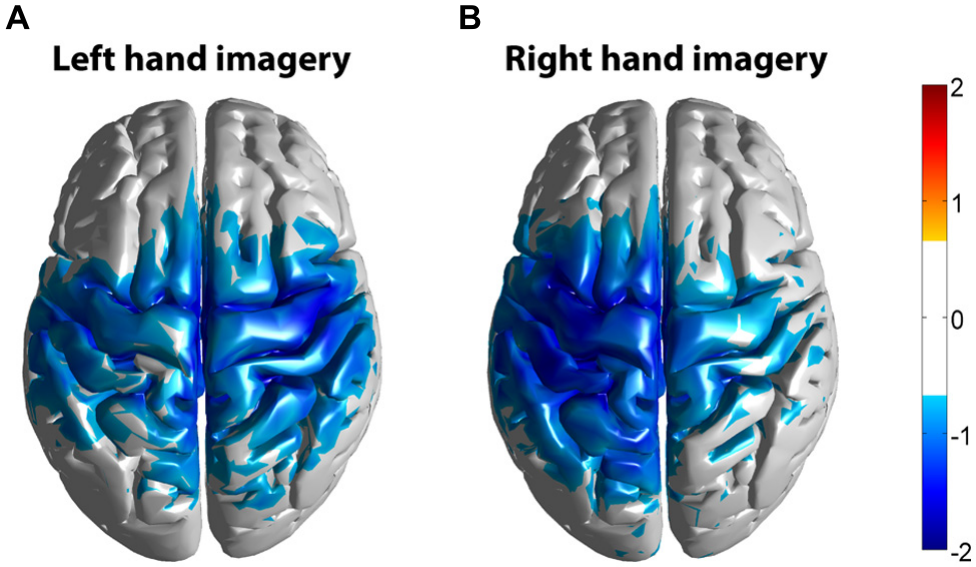
***Z*-score group average maps of beta band activity (15–35 Hz) of all subjects (*n* = 10).** Activity was mapped in Montreal Neurological Institute (MNI) space. **(A)** Averaged beta activity during left hand motor imagery. **(B)** Averaged beta activity during right hand motor imagery. The threshold for the group average was set at *Z* = 0.72, corresponding to 2.3 in a normal distribution or a 99% confidence level.

Significant HG increases were found to occur between 0.3 and 1.0 s during the task period. Baseline was selected from -1 to 0 s and the cortical distributions of relative change in the HG band were determined. Peak HG frequencies for each subject are shown in **Table 1**. Time-frequency representations were computed for each individual subject for the most significant voxel in the cortex after motor imagery cue onset (Subjects 5 and 8 shown in **Figure 9**. The remaining eight subjects are shown in **Figures 10 and 11**). Subjects showed significant HG activity in narrow bands that were centered between 70 and 90 Hz. In addition, most subjects showed a decrease in beta band activity that preceded power increases in the HG band, at the sites of HG activity. Because this result is commonly noted during motor imagery tasks and replicates a well-characterized phenomenon (Darvas et al., 2010) it is likely an additional indicator that the recorded HG activity is of genuine cortical origin. Group averages of this beta band decrease are shown as cortical representations in **Figure 12**.

**Table 1:** Peak HG frequencies between cue onset and 1 s post-cue onset during left hand (LH) and right hand (RH) motor imagery EEG sessions.

Subject	Handedness	Age	Gender	Imagined Hand	Frequency (Hz)
1	R	29	M	LH	84
				RH	87
2	R	26	F	LH	79
				RH	84
3	R	26	F	LH	85
				RH	81
4	R	30	M	LH	78
				RH	78
5	R	25	F	LH	87
				RH	90
6	L	20	F	LH	82
				RH	82
7	R	30	F	LH	85
				RH	88
8	R	20	M	LH	84
				RH	88
9	R	22	M	LH	96
				RH	89
10	R	21	F	LH	89
				RH	94

### SPATIAL CORRELATION BETWEEN fMRI BOLD RESPONSE AND EEG HG ACTIVITY

**FIGURE 13:**
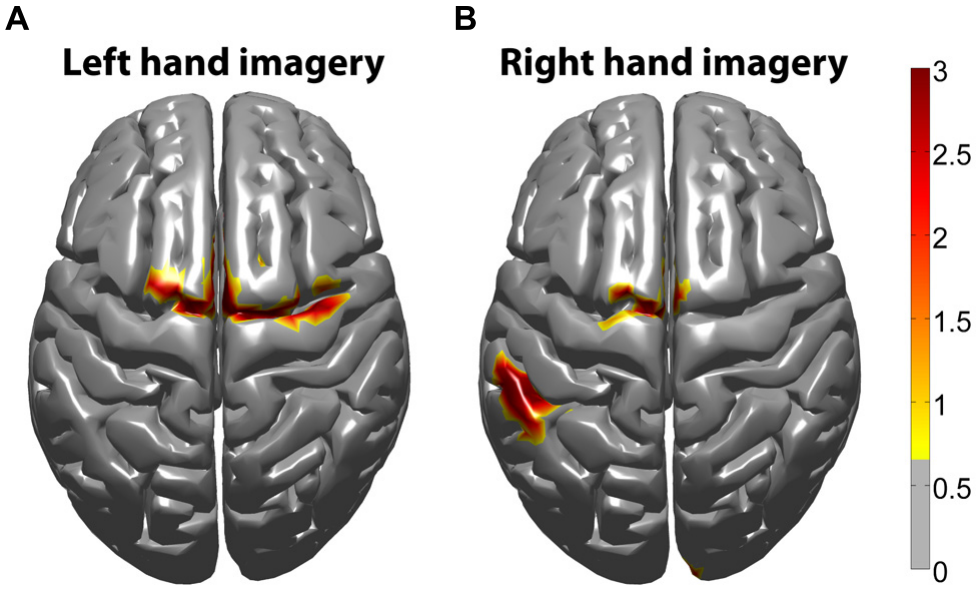
***Z*-score group average fMRI BOLD maps of all subjects (*n* = 10).** fMRI BOLD activity was averaged from all subjects in standard MNI space. **(A)** Averaged fMRI BOLD activity during left hand motor imagery. **(B)** Averaged fMRI BOLD activity during right hand motor imagery. The threshold was determined from a mixed effect model, correcting for multiple comparisons at the group level, using a Gaussian weighted cluster correction at *p* < 0.05.

The task-related fMRI BOLD changes are also shown in **Figure 6** along with the EEG source changes in HG bands on an inflated cortical surface for the imagination of movement of the left and right hands for subjects 5 and 8 (data for the remaining eight subjects are shown in **Figures 7 and 8**). Individual fMRI results showed BOLD signal increases both in the sensorimotor areas as well as additional BOLD signal increases in the surrounding cortical areas that are varied across subjects. Averages of the fMRI activity during the motor imagery of the left and right hand of all ten subjects are shown in **Figure 13**. Individual fMRI activity spatially co-localizes with the EEG locations of significant power increases in the HG band in most subjects, albeit due to the sometimes more widespread fMRI activity. We find our measure of proximity ranging from 3 to 32 mm (mean 14 mm, 8 mm SD) across all subjects and conditions (except for subject 10, left imagery, where no match exists). *p*-values range from *p* < 0.001 to *p* < 0.84, but we find across seven subjects at least one condition with *p* < 0.05 and among those two with both conditions at *p* < 0.05. Distances and *p* values are listed in **Table 2**. In most cases there is a correlation between small proximity measures and significance, but in cases where there are many fMRI clusters spread out across the cortex, the specificity of the solution remains still small.

**Table 2:** Results of the proximity analysis for all 10 subjects and left/right imagery.

Subject	Proximity – left [mm]	*p*-value left	Proximity – right [mm]	*p*-value – right
1	18	0.00	12	0.44
2	27	0.70	15	0.00
3	23	0.58	7	0.19
4	5	0.04	4	0.10
5	3	0.01	12	0.00
6	6	0.04	6	0.00
7	25	0.06	6	0.00
8	15	0.06	19	0.03
9	14	0.33	11	0.66
10	NA	NA	32	0.84

*Proximity is the average minimal distance between fMRI clusters and EEG clusters. The *p*-value is an indication of the specificity of the particular match, computed from a permutation of the proximity measure, where the fMRI clusters are spatially resampled.*

## DISCUSSION

In this study, we aimed to recover motor-imagery-related EEG changes in the HG band and examine its spatial overlap with evoked hemodynamic responses. To study this, we collected EEG and fMRI BOLD data of subjects performing a left and right hand motor imagery task in separate sessions. We compared spatial maps of fMRI BOLD signal changes to HG spectral power changes in the measured EEG potential during the motor imagery task. Both modalities were carefully co-registered to allow for direct comparison. Our results show that HG activity during motor imagery can be recovered with EEG using an individual subject’s anatomical head model and inverse modeling methods. This high frequency spectral power change has been shown to correlate directly with firing rate (Manning et al., 2009; Miller et al., 2009; Whittingstall and Logothetis, 2009) and has been demonstrated to reflect broad spectral changes across all frequencies (Miller et al., 2007, 2009). Furthermore, cortical increases in HG band activity recorded with EEG co-localized with an increase of the fMRI BOLD signal.

Taken together, these results suggest that EEG can accurately resolve spatially specific estimates of local cortical high frequency signals, potentially opening an avenue for characterizing HG signals from diverse sets of neurologically impaired populations, including various neurodegenerative disorders. Research shows that event-related oscillations in alpha, beta, gamma, delta and theta frequency bands are highly modified in pathological brains, especially in patients with cognitive impairment (Başar et al., 2012; Basar-Eroglu et al., 2012; Ozerdem et al., 2012; Yener and Bajar, 2012; Yener and Basar, 2012; Vecchio et al., 2013). Non-invasive EEG signals in these subject populations are currently only detected up to the beta frequency range, oscillations that are focally non-specific and likely represent an amalgamated signature of numerous anatomical substrates. There is growing evidence supporting the idea that disorders such as Alzheimer’s disease target specific and functionally connected neuronal networks (Reid and Evans, 2013). Thus, the non-invasive resolution of HG signals, which are believed to represent the activation of focal neural populations, may provide insights into specific neurophysiology mechanisms underlying neurodegenerative disorders such as Parkinson’s and Alzheimer’s. These gamma modulations could prove to be a useful electrophysiological biomarker to examine the pathophysiology of such neurological diseases.

### EEG HG AND fMRI CO-LOCALIZATION WITHIN THE MOTOR NETWORK

Group averaged beta decreases across the motor system during EEG recordings and increases of the fMRI BOLD signal show spatial co-localization, but individual HG results co-localize best to individual fMRI results and do not produce a coherent grand-average map. This is consistent with ECoG studies that show HG is better than beta frequencies in terms of localization on an individual basis (Hermes et al., 2012). In addition to motor cortex, EEG HG and fMRI activity were found in other cortical areas, which did not overlap when subjected to averaging to common brainspace (MNI152). This is different from our earlier work on HG activity during overt movement (Darvas et al., 2010), where a generic model was used to map HG activity, but more importantly active movement was used and the EEG signal was segmented based on actual EMG onset. This can be expected to produce a stronger and temporally better aligned HG response than motor imagery, where the only indication of onset of activity is given by the cue.

Since fMRI measures blood oxygenation as a proxy for neuronal activity and the EEG signal is a direct measure of the activity of large groups of coherently active neurons, co-localization of the two modalities is not strictly necessary. Additionally, different layers of neurons in the generation of the two signals are involved (Nunez and Silberstein, 2000), which can overlap in activation for a given task, but do not have to.

The scattered nature of the EEG localization, but also of the fMRI activity in our results could be attributable to differences in a subject’s imagery strategy. Additionally, any EEG inverse mapping method is prone to localization errors, e.g., the LCMV beamformer that we used, can introduce spurious spatial localizations, even from single “true” sources, where the time series of the reconstructed sources matches the original source, thus leading to a robust result with our trial-to-trial stability test.

### HG EEG ACTIVITY RELATED TO MOTOR IMAGERY

Frontal eye fields (FEFs) in the prefrontal cortex are thought to play a key role in the planning and execution of saccadic eye movements, as well as visual selective attention (Russo and Bruce, 1994; Bullier, 2001). MEG studies have indicated that early HG activity over the right FEF is present during saccade preparation. During saccade execution, HG activity is observed in the supplementary eye fields (SEFs), then subsequently progresses to the visual cortex and FEF bilaterally (Hinkley et al., 2011). In addition to FEF, it has been suggested that transient increases in scalp EEG gamma band power (above 30 Hz) in the parietal–occipital cortex can be linked to task-related saccadic eye movements (Reva and Aftanas, 2004; Trujillo et al., 2005; Yuval-Greenberg et al., 2008). Yuval-Greenberg et al. (2008) showed data that indicates the broadband (30–90 Hz) and transient (between 200 and 300 ms) gamma activity recorded with EEG in the parietal–occipital cortex mirrors eye movements following the display of a new image and that this gamma signal may be the consequence of associated ocular muscle artifacts engendered from miniature saccades. Invasive studies showed that saccadic ocular muscle activity might generate gamma-range artifacts in ECoG data that is confined to the medial temporal pole and is likely due to its immediate vicinity to the lateral rectus eye muscle (Jerbi et al., 2009). The results from the present study do not show significant increases in HG activity during the cued baseline period, indicating that the HG activity we see after cued motor imagery onset is related to motor imagery and not activity induced during miniature saccades.

### HG EEG AS A CONTROL SIGNAL FOR BRAIN-COMPUTER INTERFACE

In the sensorimotor areas of the cortex, motor imagery has been found to be associated with increases in the HG band, as well as decreases in the beta and mu bands. Because these cortical rhythms can be intentionally modulated by motor imagery, they have been used in non-invasive (i.e., mu and beta; Pfurtscheller et al., 2003; Pfurtscheller and Neuper, 2006) and invasive (HG, mu and beta) BCI studies as a control signal (Leuthardt et al., 2006). In addition to control signals, motor imagery-based BCIs have recently shown great potential for restoring lost function and inducing activity-dependent brain plasticity in patients suffering from paralysis due to stroke (Bai et al., 2008; Buch et al., 2008; Bundy et al., 2012). However, current non-invasive motor imagery-based BCI research has been based on the low spatial resolution offered by EEG/MEG electrodes and spatially broad mu and beta rhythms, resulting in unreliable and coarse BCI control with average information transfer rates in the range 10–20 bits/min for motor-imagery BCIs (Rao, 2013). The overall efficacy of these systems is limited due to the time it takes for the low frequency rhythm’s amplitudes to evolve, which is on the order of several hundred milliseconds (Pfurtscheller and Lopes da Silva, 1999). Ideally, a smaller response lag is desired, i.e., 100 ms or less, to ensure a more fluid alternative for device control and rehabilitation. The HG rhythms that we were able to detect in this study, with their increased spatiotemporal resolution and greater task specificity, have the potential to enhance the performance of EEG-driven orthotic and prosthetic devices by allowing the brain to interact with assistive devices on a more natural timescale. In addition, the demonstrated ability to detect HG changes oﬄine and non-invasively, a major strength of using EEG, will enable the development of paradigms that allow the neurophysiological functions in humans to be studied non-invasively on a more global scale compared to ECoG.

## Conflict of Interest Statement

The authors declare that the research was conducted in the absence of any commercial or financial relationships that could be construed as a potential conflict of interest.

## Footnotes

1 http://surfer.nmr.mgh.harvard.edu/

2 http://neuroimage.usc.edu/brainstorm

3 http://www.fmrib.ox.ac.uk/fsl

## ABBREVIATIONS

BCI: brain-computer interface
BEM: boundary element model
BOLD: blood oxygen level dependence
CAR: common average reference
EEG: electroencephalogram
EMG: electromyography
fMRI: functional magnetic resonance imaging
HG: high gamma
